# A novel Brönsted–Lewis acidic heteropoly organic–inorganic salt: preparation and catalysis for rosin dimerization

**DOI:** 10.1186/s40064-016-2098-4

**Published:** 2016-04-14

**Authors:** Bing Yuan, Congxia Xie, Fengli Yu, Xiaoying Yang, Shitao Yu, Jianling Zhang, Xiaobing Chen

**Affiliations:** State Key Laboratory Base of Eco-Chemical Engineering, College of Chemistry and Molecular Engineering, Qingdao University of Science and Technology, Qingdao, 266042 China; Beijing National Laboratory for Molecular Sciences (BNLMS), Beijing, 100190 China; College of Chemical Engineering, Qingdao University of Science and Technology, Qingdao, 266042 China

## Abstract

A novel Brönsted–Lewis acidic heteropoly organic–inorganic salt has been prepared via the replacement of protons in neat phosphotungstic acid with both organic and metal cations. This hybrid catalyst, Sm_0.33_[TEAPS]_2_PW_12_O_40_, exhibited satisfactory performance in the dimerization of rosin to prepare polymerized rosin Under optimum conditions (15.0 g rosin and 5.0 g Sm_0.33_[TEAPS]_2_PW_12_O_40_ catalyst in 18.0 mL toluene at 90 °C for 10 h), a polymerized rosin product with a softening point of 120.1 °C was obtained. In addition, the Sm_0.33_[TEAPS]_2_PW_12_O_40_ catalyst maintains excellent catalytic performance over five recycles.

## Background

Polymerized rosin has a higher softening point, lighter color, and better stability than rosin, and is harder to oxidize. It is a key ingredient in oil paints, printing ink, adhesives, perfume, and more (Cheng et al. 1996; Chen 1992). The industrial preparation of polymerized rosin, employing aqueous mineral acids such as H_2_SO_4_ or ZnCl_2_/HCl, suffers from various shortcomings, including corrosion, pollution, and difficult recovery. Some environmentally friendly catalysts, such as solid superacids (Luo and Wu 1999; Gao et al. 2007), have been used to realize the clean polymerization of rosin. However, despite their superior separation, solid superacids exhibit insufficient recycling performance due to their uneven and vulnerable active components.

**Scheme 1:**
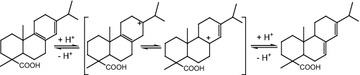
The isomerization of fir-type resin acids towards abietic-type resin acids

**Scheme 2:**
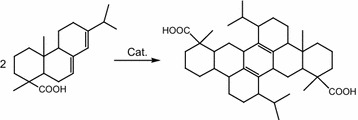
The dipolymerization of abietic-type resin acids

Acid-functionalized ionic liquids, a class of catalyst with the advantages of both aqueous and solid acids, have been applied successfully in many acid-catalyzed reactions (Paun et al. 2008; Fang et al. 2008; Hoang et al. 2005), including the polymerization of rosin (Liu et al. 2005, 2008). Abietic-type resin acids in gum rosin, which contain conjugated double bonds, undergo polymerization readily, but fir-type resin acids, having more steric hindrance, cannot. Brönsted acids are generally considered more apt to promote the isomerization of fir-type resin acids towards abietic-type resin acids (Scheme 1), while Lewis acids favor the dipolymerization of abietic-type resin acids (Scheme 2) (Cheng et al. 1996). As a result, acid catalysts comprising both Brönsted and Lewis acidity would exhibit a more outstanding catalytic performance in the preparation of polymerized rosin. We previously synthesized (3-sulfonic acid)-propyl-3-methylimidazoliume (and triethylammonium) chlorozincates and demonstrated their good catalytic efficiency and recycling performance in the polymerization of rosin, which can be attributed to the Brönsted–Lewis acidity of the ionic liquids and liquid–liquid two-phase process (Liu et al. 2008, 2009). However, using these catalysts is inconvenient due to their lengthy synthetic cycles and high viscosity. Most importantly, the Lewis acidity of the metal chlorides in the anions would be difficult to maintain due to stability issues, similar to aluminum chloride acid salt ionic liquids.

For the past few years, types of heteropoly organic salt catalytic materials have called attention for their potential water tolerance, acidity and self-separation performance (Leng et al. 2009a, b, 2012; Li et al. 2011, 2014; Shimizu et al. 2009; Sun et al. 2012; Zhou et al. 2014). It has been found that heteropoly anions with high charge numbers in these materials lead to higher melting points than conventional ionic liquids (Yuan et al. 2014). Furthermore, based on the high charge numbers of heteropoly anions, sulfated organic cations with Brönsted acidity and metal cations with Lewis acidity can act together as counterions to heteropoly anions, establishing novel Brönsted–Lewis acidic heteropoly organic–inorganic salts (Yu ST 2013). Herein, we report a heteropoly organic–inorganic catalyst, Sm_0.33_[TEAPS]_2_PW_12_O_40_, with Brönsted–Lewis acidity, which has different performances for melting point, solubility and acidity with both heteropoly compounds and ionic liquids. Moreover, the dimerization of rosin catalyzed by Sm_0.33_[TEAPS]_2_PW_12_O_40_ as a solid acid has been carried out to achieve an environmentally friendly process for polymerized rosin.

## Experimental

### Materials and methods

Analytical grade H_3_PW_12_O_40_ was dried at 180 °C. All other chemicals were of analytical grade and used without further purification. The ^1^H-NMR spectra of the catalyst and intermediates were recorded with a 500 MHz Bruker spectrometer in D_2_O. FT-IR spectra for catalyst samples (the Py-IR sample was mixed with pyridine (2:1, *v*/*v*) for 24 h prior to measurement) on KBr discs were recorded on a Nicolet iS10 FT-IR instrument. Melting points were measured using a conventional method on an X-4 type micro melting point apparatus. TG analysis was performed with a NETZSCH-TG 209 F1 Libra instruments in dry N_2_ at a heating rate of 20 °C/min from 30 to 800 °C.

The acidity of the prepared catalysts was determined by potentiometric titration (Shi and Pan 2008; Vazquez et al. 2000). A mixture containing the sample (0.5 g) and acetonitrile (30 mL) was mixed at the stable potential before being titrated with *n*-C_4_H_9_NH_2_ solution (0.05 mol/L in acetonitrile). The initial and jump potential values were measured by a pH meter to identify the acid strength and total acid amount in catalyst samples.

### Catalysts

Contrastive catalyst, [TEAPS]_3_PW_12_O_40_ and H_2_[TEAPS]PW_12_O_40_, were prepared according to the literature (Leng et al. 2009b). Analogously, equimolar triethylamine and 1,3-propanesultone (0.10 mol) were dissolved in 80 mL ethyl acetate and stirred at 50 °C for 24 h under nitrogen atmosphere. The obtained white precipitate, 3-(triethylammonio)propane sulfonate, was filtered, washed with ethyl acetate and dried at 100 °C for 6 h. Next, a solution of intermediate 3-(triethylammonio)propane sulfonate (0.008 mol) and Sm(NO_3_)_3_·6H_2_O (0.0013 mol) in water was dropped into another aqueous solution of H_3_PW_12_O_40_ (0.004 mol). The mixture was stirred at room temperature for 24 h, distilled to remove water, and washed with ethyl acetate. Finally, the obtained Sm_0.33_[TEAPS]_2_PW_12_O_40_ solid was dried in a vacuum at 80 °C for 6 h (Leng et al. 2009b; Ramesh Kumar et al. 2012). Catalyst Sm_0.66_[TEAPS]PW_12_O_40_ was prepared by a similar method to that outlined above, using alternative materials proportion.

### Dimerization of rosin

In batch experiments, heteropoly organic–inorganic salt catalyst (5.0 g) was added to a round-bottomed flask contained toluene (18.0 mL) and dissolved rosin (15.0 g). The resulting reaction mixture was stirred vigorously at 90 °C for 10 h and then cooled to room temperature. The solid catalyst was removed by centrifugation and directly reused without further treatment. The reaction solution, from which the toluene solvent had been separated, was distilled under low pressure (2 mmHg) at 260–270 °C (system temperature) and 180–210 °C (steam outlet temperature) for 30 min to remove low softening point materials, such as rosinol and some unpolymerized rosin, and obtain the polymerized rosin product. The ring and ball softening points of the products were determined by SYD-2806G numerical control asphalt softening point tester.

## Results and discussion

### Characterization of hybrid catalysts

**Fig. 1:**
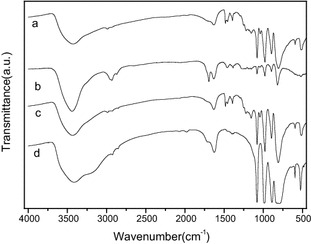
FT-IR spectra for hybrid catalysts. *a* Sm_0.33_[TEAPS]_2_PW_12_O_40_. *b* Reused Sm_0.33_[TEAPS]_2_PW_12_O_40_. *c* H_2_[TEAPS]PW_12_O_40_. *d* H_3_PW_12_O_40_

**Fig. 2:**
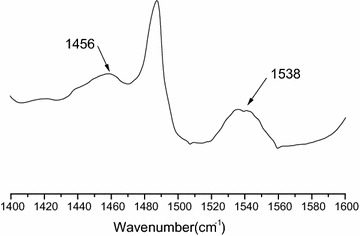
Py-IR spectra for Sm_0.33_[TEAPS]_2_PW_12_O_40_

Similar patterns were observed in the ^1^H-NMR (500 MHz, D_2_O) spectra of Sm_0.33_[TEAPS]_2_PW_12_O_40_, Sm_0.66_[TEAPS]PW_12_O_40_, [TEAPS]_3_PW_12_O_40_, H_2_[TEAPS]PW_12_O_40_ and their intermediate 3-(triethylammonio)propane sulfonate [δ 1.17 (t, 9H), 2.02 (m, 2H), 2.87 (t, 2H), 3.23 (m, 8H)], which supported the correct structure of the organic cations (Leng et al. 2009b). The FT-IR spectrum of Sm_0.33_[TEAPS]_2_PW_12_O_40_ is shown in Fig. 1, in comparison with those of reused Sm_0.33_[TEAPS]_2_PW_12_O_40_, H_2_[TEAPS]PW_12_O_40_, and neat H_3_PW_12_O_40_. It was observed that Sm_0.33_[TEAPS]_2_PW_12_O_40_, reused Sm_0.33_[TEAPS]_2_PW_12_O_40_, and H_2_[TEAPS]PW_12_O_40_ had four featured peaks similar to those of H_3_PW_12_O_40_ [1080 (P–O), 984 (W=O), 889 (W–O_b1_–W) and 806 cm^−l^ (W–O_b2_–W)], which were assigned to the Keggin structure. Different degree shifts (around 1079, 981, 897 and 806 cm^−l^) and lower peak intensities compared to H_3_PW_12_O_40_ confirmed that the formation of new heteropoly organic–inorganic salts had been achieved without breaking the Keggin structure. In addition, featured peaks at 2987, 1485, 1394, 1127 and 1156 cm^−1^ of TEAPS^+^ (Liu et al. 2008; Leng et al. 2009b) also appeared in the spectra of the hybrid catalysts. The above results indicated that both TEAPS^+^ and PW_12_O_40_^3−^ were combined in all of the hybrid catalysts. Moreover, the Py-IR spectrum illustrated in Fig. 2 indicates the existence of both Brönsted acid sites (1538 cm^−1^) and Lewis acid sites (1456 cm^−1^) in Sm_0.33_[TEAPS]_2_PW_12_O_40_, which proved the presence of Brönsted–Lewis acidity derived from organic cations and Sm^3+^.

Table 1 shows the basic properties of the designed Sm_0.33_[TEAPS]_2_PW_12_O_40_ catalyst and similar substances. It was found that the introduction of Sm^3+^ lead to samples having properties similar to inorganic salts (melting points >300 °C) and quite different to those of heteropoly ionic liquids [TEAPS]_3_PW_12_O_40_ and neat H_3_PW_12_O_40_. Concerning acidity, a lower acid strength and total acid amount was observed in hybrid samples with metal cations compared with [TEAPS]_3_PW_12_O_40_ and H_3_PW_12_O_40_. Besides, the poor solubility of Sm_0.33_[TEAPS]_2_PW_12_O_40_ in both water and organic solvent toluene forced it to be used as a solid catalyst in the prospective reaction.

**Table 1:** Properties of hybrid catalysts

Samples	Feature	Melting point (°C)	Solubility (25 °C)		Acid strength (mV)	Total acid amount (mmol/g)
			Water	Toluene		
Sm_0.33_[TEAPS]_2_PW_12_O_40_	White powder	>300	Almost not	Not	679	0.428
Sm_0.66_[TEAPS]PW_12_O_40_	White powder	>300	Almost not	Not	676	0.148
[TEAPS]_3_PW_12_O_40_	White powder	178	Almost not	Not	718	0.873
H_3_PW_12_O_40_	Faint yellow/white crystal	95	Yes	Not	702	0.791

**Fig. 3:**
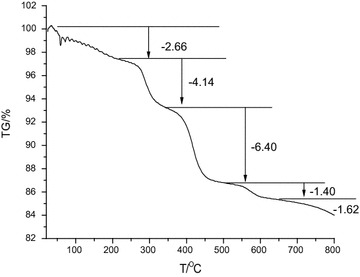
TG profiles of Sm_0.33_[TEAPS]_2_PW_12_O_40_

Figure 3 shows that Sm_0.33_[TEAPS]_2_PW_12_O_40_ loses unbound and crystal water (calculated weight loss: 2.66 %) between 30 and 200 °C. Moreover, the decomposition peaks of the organic cation matrix and sulfonic group were observed at 290 and 420 °C, respectively. The TG profile indicated that Sm_0.33_[TEAPS]_2_PW_12_O_40_ was quite stable below 200 °C.

### Catalytic performances of Sm_0.33_[TEAPS]_2_PW_12_O_40_ in the dimerization of rosin

Table 2 shows that a product with a similar softening point and acid value to gum rosin was obtained without catalyst. This was also observed in the reaction catalyzed by H_3_PW_12_O_40_, having high acid strength, indicating that Brönsted acidity derived from the antiprotons of H_3_PW_12_O_40_ had almost no effect on the promotion of rosin dimerization under the solid acid catalyst conditions. In addition, the Brönsted acidity derived from sulfonic acid groups in the organic cations alone did not have the necessary catalytic ability for dimerization, resulting in a product with 103.7 °C softening point. When both Sm^3+^ and organic cations containing sulfonic acid group [TEAPS]^+^ were co-introduced to the structure of a heteropoly compound, Brönsted–Lewis double acidity (Fig. 2) and, consequently, a high catalytic performance were achieved (Table 2). In particular, a favorable polymerized rosin product with a softening point of 120.1 °C [higher than the result of Reference Liu et al. (2009) but lower than that of reference Liu et al. (2008)] was obtained by Sm_0.33_[TEAPS]_2_PW_12_O_40_, a hybrid catalyst with proper Brönsted and Lewis acidity proportions. However, the Lewis acid sites of Sm^3+^ in Sm_0.33_[TEAPS]_2_PW_12_O_40_ were surrounded by more bulky phosphotungstic acid radicals and organic cations than those of Sm_0.66_[TEAPS]PW_12_O_40_ and the Brönsted–Lewis Acidic ionic liquids reported in References (Liu et al. 2008, 2009). For this reason, the decarboxylated conjugated resin acids with lower steric hindrance attached more easily to Sm^3+^ than the conjugated resin acids without decarboxylation, which accounted for the lower acid value of products obtained from the Sm_0.33_[TEAPS]_2_PW_12_O_40_ catalyst (Liu et al. 2008, 2009).

**Table 2:** Catalytic dimerization performance of hybrid catalysts

Catalysts	Softening point (°C)	Acid value (mg/g)
Gum rosin	80.4	164.0
Blank	88.3	165.0
H_3_PW_12_O_40_	86.5	137.8
[TEAPS]_3_PW_12_O_40_	103.7	134.7
Sm_0.66_[TEAPS]PW_12_O_40_	111.9	147.9
Sm_0.33_[TEAPS]_2_PW_12_O_40_	120.1	111.8

Reaction conditions: catalyst 5.0 g, rosin 15 g, toluene 18 mL, 90 °C, 10 h

The solid catalyst, Sm_0.33_[TEAPS]_2_PW_12_O_40_, precipitated to the bottom of the reactor after the reaction, allowing separation from the solution via simple centrifugation. The separated Sm_0.33_[TEAPS]_2_PW_12_O_40_ catalyst could be reused several times without treatment (see Table 3). The polymerized rosin products with high softening points (above 120 °C) were obtained even after reusing the catalyst five times, which was attributed to the concerted catalysis of Brönsted and Lewis acid sites and their covalent or ionic bonding pattern in the body of the catalyst in the solid–liquid catalytic system. The FT-IR spectrum of reused Sm_0.33_[TEAPS]_2_PW_12_O_40_, shown in Fig. 2, also indicates that no apparent structural change had taken place in the catalyst during use.

**Table 3:** Catalytic reusability of Sm_0.33_[TEAPS]_2_PW_12_O_40_ for the rosin dimerization

Catalyst recycle times	Softening point (°C)	Acid value (mg/g)
0	120.1	111.8
1	114.3	125.8
2	115.0	120.3
3	126.1	124.5
4	120.4	93.7
5	120.3	96.0

Reaction conditions: catalyst 5.0 g, rosin 15 g, toluene 18 mL, 90 °C, 10 h

## Conclusion

A novel heteropoly organic–inorganic salt with Brönsted–Lewis double acidity, Sm_0.33_[TEAPS]_2_PW_12_O_40_, was prepared via the replacement of protons in neat phosphotungstic acid with both organic cations containing sulfonic acid groups and metal Sm^3+^ cations. As a solid acid catalyst, this environmentally benign Brönsted–Lewis double acidic hybrid enables an effective catalytic performance in the dimerization of rosin to afford polymerized rosin products with a softening point above 115 °C. Moreover, the catalyst also exhibited reasonable reuseability, demonstrated by a five-run recycling test.
